# Obesity, Early Life Gut Microbiota, and Antibiotics

**DOI:** 10.3390/microorganisms9020413

**Published:** 2021-02-17

**Authors:** Alyssa T. Wilkins, Raylene A. Reimer

**Affiliations:** 1 Faculty of Kinesiology, University of Calgary, Calgary, AB T2N 1N4, Canada; alyssa.wilkins@ucalgary.ca; 2 Department of Biochemistry & Molecular Biology, University of Calgary, Calgary, AB T2N 1N4, Canada

**Keywords:** infancy, obesity risk, gut microbiota, early-life, antibiotics

## Abstract

Obesity is a major public health problem that continues to be one of the leading risk factors for premature death. Early life is a critical period of time when the gut microbiota and host metabolism are developing in tandem and significantly contribute to long-term health outcomes. Dysbiosis of the gut microbiota, particularly in early life, can have detrimental effects on host health and increase the susceptibility of developing obesity later in life. Antibiotics are an essential lifesaving treatment; however, their use in early life may not be without risk. Antibiotics are a leading cause of intestinal dysbiosis, and early life administration is associated with obesity risk. The following review explores the relevant literature that simultaneously examines antibiotic-induced dysbiosis and obesity risk. Current evidence suggests that disruptions to the composition and maturation of the gut microbiota caused by antibiotic use in early life are a key mechanism linking the association between antibiotics and obesity. Without compromising clinical practice, increased consideration of the long-term adverse effects of antibiotic treatment on host health, particularly when used in early life is warranted. Novel adjunct interventions should be investigated (e.g., prebiotics) to help mitigate metabolic risk when antibiotic treatment is clinically necessary.

## 1. Introduction

Obesity is a global public health problem, for which the prevalence of worldwide obesity has nearly tripled since 1975 [1]. Current estimates by the World Health Organization indicate that 650 million adults have obesity, 340 million children ages 5–19 are overweight or have obesity, and 38.2 million children under the age of 5 have are overweight or have obesity [1]. Together, overweight and obesity are the fifth leading cause of mortality globally, while other top risk factors, including high blood pressure, elevated blood glucose, and physical inactivity either contribute to or result from excess body weight [2]. 

Obesity is known to be a major health challenge in high-income countries, however, in 2013, 62% of the population with obesity resided in low- or middle-income countries [3]. A rapid increase in obesity rates has recently been observed in low- and middle-income countries that have historically seen higher rates of undernourishment [4,5]. With obesity rates rising worldwide, there are now more cases of obesity globally than underweight [1]. 

Obesity in childhood and adolescence is associated with the risk of remaining overweight in later life and predisposes an individual to long term health consequences [6,7]. Obesity is associated with comorbidities, including type 2 diabetes (T2D), cardiovascular disease, hypertension, non- alcoholic fatty liver disease (NAFLD), dyslipidemia, certain cancers, osteoarthritis, and obstructive sleep apnea [4,6]. In the past, many of these diseases have occurred exclusively in adulthood, but with the rise in childhood obesity, comorbidities are becoming more prevalent in children and adolescents [6].

## 2. Obesity Etiology 

The etiology of obesity encompasses complex interactions between environmental and genetic factors that result in an energy imbalance, leading to the deposition of excess adipose tissue. The rapid rise in obesity rates in recent years is often attributed to the global transition toward an obesogenic environment [8]. An obesogenic environment encourages overconsumption of energy-dense foods in conjunction with reduced physical activity levels, leading to a positive energy balance [6,8]. Although individuals may be exposed to a similar obesogenic environment, large variations in weight still occur, demonstrating the potential role genetics play in the development of obesity. Obesity-linked genes influence weight through alterations in key metabolic pathways, including, for example, appetite regulation [8]. While several genes linked to the regulation of body weight have been identified, genetic susceptibility accounts for only a small percentage of the variance in body weight, suggesting that other environmental factors play a large role [9].

Prenatal and postnatal development are critical periods of life when perturbations and early life experiences are strong predictors of childhood and adulthood obesity development [8,10]. During gestation, the maternal environment can influence obesity risk as the fetus adapts to the intra-uterine environment it is exposed to, in part through epigenetic regulation [10,11]. During fetal development, exposure to gestational diabetes, maternal smoking, high gestational weight gain, intrauterine growth restriction, and maternal obesity are associated with an increased incidence of obesity in childhood [10,12,13]. Other important risk factors in early life include birth size, rapid and/or excessive weight gain during infancy, early onset of the adiposity rebound, and breast-feeding exposure [6,10,12]. 

Together with genetic and environmental factors, there is growing evidence that the gut microbiota plays an important role in the development of obesity. An imbalance or disrupted microbial environment termed gut microbiota dysbiosis, influences obesity pathogenesis by impacting energy harvest, nutrient metabolism, inflammatory pathways and the gut-brain axis [5,8,14].

## 3. Gut Microbiota

The gut microbiota encompasses all of the microorganisms that colonize the gut from the esophagus through to the rectum, including bacteria, viruses, archaea, and eukarya [14,15,16]. The gut microbiome refers to the collective genome of all the microorganisms in the intestinal tract. It was previously believed that over 10^14^ microorganisms colonize the gastrointestinal tract, exceeding the number of human cells in the host by approximately ten times [14]. Updated estimates now suggest the ratio of human cells to bacterial cells to be approximately 1:1 [14]. The human gut microbiota is predominantly comprised of two main phyla, *Bacteroidetes* and *Firmicutes*, which account for ~90% of the gut microbiota [17]. Other dominant phyla include *Actinobacteria*, *Proteobacteria*, *Fusobacteria*, and *Verrucomicrobia* [17]. The intestinal microbiota is characterized by its abundance, diversity, richness, and composition. Microbial colonization differs throughout the gastrointestinal tract due to variations in the gut environment, such as nutrient and oxygen availability and pH [9]. The proximal gut is dominated by aerobic bacteria, while the large intestine is colonized by predominantly anaerobic microbes [9] and houses approximately 70% of all microbes in the body [15]. The symbiotic relationship formed between the host and the microbes plays a vital role in early life development, lifelong health, and an array of disease states [15,18]. 

### 3.1. Gut Microbiota Development

Recent studies suggest that the prenatal gut may not be sterile at birth as a result of small-scale colonization occurring in utero, however, this hypothesis remains controversial [14,15,16]. In studies using molecular techniques and high throughput sequencing, microbial populations have been detected in the placenta, amniotic fluid, and meconium [18,19,20,21]. For example, one study showed that placental tissue from 300 women had a lowly abundant microbiome that included *Bacteroides* spp., *Prevotella tannerae*, *Fusobacterium* spp. and *Escherichia coli*, among others [19]. In another study, *Enterobacter* and *Escherichia*/*Shigella* were found to be the most prevalent genera detected in both placenta and amniotic fluid in full-term, healthy mothers [22]. Some researchers have attributed premature birth and low birth weight in full-term neonates to variations in the placental microbiome [19,20]. Despite these studies, the evidence for in utero colonization is currently considered extremely weak as reviewed by Perez-Munoz et al. [16]. In their critical assessment of the evidence for and against a sterile womb, the authors consider the anatomical, physiological, and immunological barriers provided by the placenta, which would uphold a sterile fetal environment [16]. In addition to limitations with the methodological approaches used to show bacteria in the fetal environment, the authors suggest that the ability to derive germ free animals through aseptic transfer of the whole uterus provides compelling evidence for the sterile womb [16]. While the placenta likely does not harbor a microbiota, it is clear that maternal diet and maternal obesity during pregnancy do influence the gut microbiota of their offspring during birth and early postnatal life [23,24]. In a Canadian birth cohort study, Tun et al. [25] reported an association between maternal overweight/obesity during pregnancy and offspring overweight/obesity susceptibility at 1 and 3 years of age that was accompanied by enrichment of infant microbiota with *Lachnospiraceae* and greater species richness and diversity within the *Firmicutes* phylum. From an American cohort study, Chu et al. [23] demonstrated that the neonate microbiota is significantly affected by a maternal high fat diet during pregnancy, independent of maternal body mass index (BMI).

The microbiome in early life undergoes rapid colonization and is highly susceptible to environmental factors that affect the gut microbiota’s composition and function [26]. Facultative anaerobes are the first microbes to colonize healthy neonates and generally remain dominant until they use up the oxygen in the gut and pave the way for more a complex microbial community [27]. Factors, such as mode of delivery, play a major role in influencing the early colonizers in the infant gut. During a vaginal delivery, an infant’s gut is colonized by organisms acquired from maternal vaginal microbes [28] and are dominated by *Lactobacillus*, *Prevotella*, and *Sneathia* spp. In contrast, infants delivered by caesarean section have a microbiota that reflects pioneer microbiota derived from the maternal skin and environmental microbes, and are dominated by *Staphylococcus*, *Corynebacterium*, and *Propionibacterium* spp. [28]. Although the differences in microbial composition due to delivery mode fade over time, these early life variations remain linked to chronic disease risk in later life, such as obesity. For example, Kalliomaki et al. [29] showed that lower abundance of bifidobacteria and enrichment in *Staphylococcus aureus* in fecal samples in infancy were associated with overweight at seven years of age. In a systematic review and meta-analysis, birth by caesarean section was associated with a higher risk of developing obesity in childhood than vaginal birth [30]. 

Feeding mode is another major influence impacting the development of the microbiome in early life. Substantial differences in dominant microbial organisms have been reported between infants who are breastfed compared to formula-fed including increased *Bifidobacterium longum* and *Lactobacillus johnsonii*/*L. gasseri* in breastfed and increased *Clostridium difficile* and *Citrobacter* spp. in formula-fed [26]. Breast milk contains nondigestible oligosaccharides, which are utilized by the gut microbiota and selectively stimulate the growth of various members of the *Bifidobacterium* genus as well as *Bacteroides* and *Lactobacillus* species (reviewed in [31]). Formula-fed infants have a more diverse microbiota, but a lower number of total bacteria [32]. Formula feeding has also been associated with a more rapid maturation of the microbiome towards an adult state and a higher abundance of inflammatory-type microbiota [26]. The microbiome is also affected by other environmental factors including geographical location and gestational age, as well as genetics [33,34,35]. 

A neonate’s gut microbiota is originally unstable, lacking diversity, and is predominantly colonized by *Bifidobacterium* [33,34]. During the first few years of life, the microbiota matures and diversifies, leading to an intestinal microbiota that is functionally and structurally comparable to an adult’s, typically by the age of 3 [26]. The microbiota progressively stabilizes throughout adolescence and shares a core microbiota with adults but still retains a higher abundance of *Bifidobacterium* and *Clostridium* [36,37]. In adulthood, long-term diet plays a major role in shaping the gut microbiota, and has been associated with varied enterotype classifications [38]. Short-term dietary modifications, particularly fiber and fat content, can alter the composition of the gut microbiota within 24 h of the diet’s initiation, but researchers showed that the enterotype remained largely stable during a ten day study [39]. The adult gut microbiota persists until approximately 70 years of age, when it once again becomes more unstable and the core microbiota shift to include increased abundance of potentially pro-inflammatory commensals and a decrease in beneficial microbes, such as members of *Verrucomicrobia* [40]. 

### 3.2. Gut Microbiota Function 

The commensal gut microbiota performs several vital functions within the host, making the intestinal microbiome an essential factor influencing human health and metabolism. The gastrointestinal tract is the largest immune organ in the body where approximately two-thirds of immune tissue and three-quarters of immunoglobulin producing cells reside [41]. Within the gastrointestinal tract, the gut microbiota plays an integral role in the development and programming of the immune system, influencing long term health and disease risk [42,43,44]. The gut microbiota plays a role in the maintenance of the intestinal mucosal barrier and protection against pathogens by preventing pathogenic bacteria and endotoxin translocation [45,46,47]. The gut microbiota also assists with essential metabolic processes including the metabolism of nutrients, drugs, and dietary toxins [9]. Within the large intestine, dietary fibers, which cannot be digested by host enzymes, are fermented by bacteria into short-chain fatty acids (SCFA), which can normally supply up to 10% of daily energy intake. In obesity, it is proposed that the gut microbiota are more efficient at extracting energy from the diet with Turnbaugh et al. showing that fecal samples of obese mice had increased SCFA concentrations and significantly less energy remaining in their feces compared to lean littermates even while consuming a standard healthy chow diet [48]. SCFA are involved in biological processes including hepatic gluconeogenesis [49] and cholesterol biosynthesis [50]. Other critical functions of the gut microbiota include influencing infant growth [51], synthesizing essential vitamins and micronutrients, transforming cholesterol into bile acids [52], and impacting the development of the hypothalamic-pituitary-adrenal system [53].

### 3.3. Gut Microbiota and Obesity 

Approximately half of the functional gut microbiota, known as the core microbiome, is similar in all humans, but each individual’s microbiome is unique [54]. The large inter-individual variation in the gut microbiota makes it difficult to define a distinct healthy or obese microbiota. Obesity, however, has been associated with an altered microbial profile that includes reduced diversity [54], increased abundance of pro-inflammatory bacteria, and decreased abundance of anti-inflammatory bacteria [55]. The ratio between the two most abundant phyla in the human microbiota, *Firmicutes* and *Bacteroidetes*, was considered as a potential biomarker for obesity early on but the characterization of the gut microbiota in obesity has since expanded. While numerous studies on animals and humans have found lower *Bacteroidetes* and a greater abundance of *Firmicutes* in participants with obesity, compared to their lean counterparts [56,57,58,59,60], other studies have reported conflicting results [61,62,63,64]. Inconsistencies within the literature are likely due to small sample sizes, inter-individual differences, and variations in the methods used to assess the gut microbiota. At the genera level, the abundance of *Bifidobacterium*, *Christensenellaceae*, *and Akkermansia* has been shown to be lower in obesity compared to lean individuals [65]. In contrast, the abundance of *Lactobacillus* has also been shown to be increased in individuals with obesity, although it is important to note that different species within this genus have contrasting effects on body weight [66]. A meta-analysis conducted by Million et al. found that *Lactobacillus acidophilus* was associated with weight gain while *Lactobacillus gasseri* was associated with weight loss in humans and animals [67]. 

#### 3.3.1. Energy Extraction and SCFA

Germ-free (GF) animal and microbial transplant studies have demonstrated that the presence and composition of the gut microbiota can play a causal role in obesity pathology. GF mice, which are devoid of microbiota and raised in a sterile environment, were leaner in comparison to mice with a conventional microbiota, despite their greater chow consumption [68]. After transplantation of cecal microbiota from conventional mice into GF mice, GF mice had a marked increase in body fat, in conjunction with reduced chow consumption, elevated leptin levels, increased fasting glucose, and insulin resistance [68]. When exposed to a western diet (i.e., diet high in sugar and fat), conventional mice experienced pro-inflammatory processes that progressed into weight gain and obesity [69]. Alternatively, body weight, fat mass, plasma insulin, and glucose levels were not significantly affected in the GF mice when they consumed the western diet [69]. Obese and lean phenotypes can be transferred from mice and humans via the microbiota into GF mice, leading to alterations in weight gain and adiposity [48,70,71]. GF mice receiving fecal microbiota transplants from subjects with obesity gained more fat than those conventionalized with lean donor’s microbiota, confirming the important role that the gut microbiota plays in obesity development [48]. Studies with GF animals have helped to increase our understanding of how gut microbiota colonization or lack thereof influences the host’s ability to harvest energy. 

Bacteria in the large intestine ferment nondigestible polysaccharides to produce SCFAs [72], which in turn provide energy to the host and regulate appetite and fat storage, thereby influencing obesity susceptibility [73,74]. The major SCFAs produced in the gut include acetate, propionate, and butyrate, which all influence metabolic functioning [71]. SCFAs confer many health benefits to the host by regulating metabolic processes in the gut, including gut motility, absorption, immune function, intestinal mucosa function as well as insulin, glucose, and lipid homeostasis in the body [74,75,76,77,78,79,80]. It has been reported that butyrate and propionate reduce appetite via their role in the stimulation of gut-derived appetite hormones [74], while acetate has been associated with brown adipose tissue thermogenesis and the browning of white adipose tissue [73]. Collectively, these functions implicate SCFAs in the regulation of body weight. In the context of obesity, however, there is debate regarding the impact of SCFAs on energy balance. On the one hand, evidence from humans and mice suggests that the obesity-associated microbiota possess a greater capacity to harvest energy from their diet, with SCFAs thereby serving as a source of energy and contributing to energy storage [48,81]. On the other hand, SCFAs can activate free fatty acid receptors that increase secretion of gut hormones that reduce food intake [82]. 

SCFAs are ligands for G-protein-coupled receptors, free fatty acid receptors 2 and 3 (FFAR2, FFAR3), which are expressed on intestinal cells and other cells in the host known to regulate various physiological and cellular functions [82]. FFAR2 deficient mice fed a high-fat diet have reduced fat mass and increased lean mass, making them resistant to diet-induced obesity [83]. This finding suggested that FFAR2’s regulation of adipose tissue expansion is at least in part influenced by the gut microbiota and their production of SCFAs [83]. FFAR3 also influences fat mass by increasing adipogenesis and inhibiting lipolysis as well as regulating the release of peptide tyrosine tyrosine (PYY), a satiety hormone that inhibits small intestine motility and suppresses appetite [84,85]. Via FFAR2/3, SCFAs also control the release of another satiety hormone glucagon-like peptide-1 (GLP-1), which regulates glucose and lipid homeostasis and suppresses appetite [74]. 

#### 3.3.2. Bile Acids

The gut microbiota influences cholesterol and bile acid (BA) metabolism [86]. Within the liver, cholesterol is used to synthesize primary BAs which are then secreted into the gut lumen and are transformed into secondary BAs by bacteria, particularly clostridia [87]. Secondary BAs are metabolic regulators that influence the metabolism of lipids, glucose homeostasis, and insulin sensitivity [87]. Primary and secondary BAs activate the nuclear farnesoid X receptor (FXR), which regulates a number of processes involved in lipid metabolism and central regulation of energy and glucose metabolism [88]. BAs can also activate the G-protein-coupled bile acid receptor, TGR5, which modulates glucose homeostasis by increasing GLP-1 release [88]. TGR5 appears to mediate bile acid-induced stimulation of energy expenditure in brown adipose tissue and skeletal muscle [89]. Bile salt hydrolase (BSH) is an enzyme produced by intestinal bacteria that catalyzes the hydrolysis of conjugated BAs into unconjugated BAs thereby regulating lipid and cholesterol metabolism and influencing host weight gain and adiposity [90]. Increasing BSH activity in mice was shown to significantly reduce weight gain, serum cholesterol, and liver triglycerides [90]. Given the diverse signaling functions of BAs and their activation of FXR and TGR5, research is underway to determine if BA-related targets could be developed for the treatment of obesity [91].

#### 3.3.3. Inflammation and Immunity

Obesity is associated with chronic low grade-inflammation and alterations in immune function, which can arise from an obesogenic gut microbiota [92]. Consumption of a high-fat diet (HFD) can lead to metabolic endotoxemia, which is mediated by translocation of lipopolysaccharides (LPS), a component of the outer membrane of Gram-negative bacteria, into systemic circulation [93]. Consuming a HFD downregulates the expression of epithelial tight junction proteins such as occludin and zonula occludens-1 (ZO-1), which in turn increases intestinal permeability and allows LPS to penetrate the intestinal epithelium [93]. The presence of LPS in the bloodstream leads to the activation of innate immunity and inflammatory pathways via toll-like receptor-4 (TLR-4) that in turn reduces insulin sensitivity [94]. Mice infused with LPS experience weight gain, increased adiposity, and show markers of elevated risk of T2D and cardiovascular disease [93].

### 3.4. Gut Microbiota Dysbiosis and Antibiotics

The microbes that reside in the human gastrointestinal tract contribute significantly to host health when a symbiotic relationship exists [95]. If the microbial community becomes disturbed (i.e., gut dysbiosis) it may contribute to the development of a variety of disease states [95]. Many non-communicable diseases have been associated with microbiota dysbiosis, including allergies, asthma, autism spectrum disorder, T2D, irritable bowel syndrome, and obesity [95]. 

During the first 3 years of life, as the gut microbiota develops towards a mature state, it plays a crucial role in the establishment of the immune system and the maintenance of gastrointestinal homeostasis [95]. Perturbations in early life that affect the composition, maturation, and function of the gut microbiota can lead to adverse health outcomes later in life, such as obesity [18]. It is commonly agreed upon that obesity is associated with dysbiosis of the gut microbiota and that reduced diversity and richness of an obesogenic microbiota is associated with inflammation, intestinal barrier dysfunction, and increased energy harvest [38]. It has been demonstrated that deviations in the composition of the gut microbiota in early life precede the development of obesity and overweight in childhood, suggesting that the obesogenic gut microbiota may be initiated in early life [29,96,97,98]. Kalliomaki et al. reported that a reduced abundance of bifidobacteria and a greater abundance of *Staphylococcus aureus* in infant fecal samples was associated with being overweight at the age of seven years [29]. Additionally, Luoto et al. found that reduced bifidobacteria in fecal samples at three months of age was associated with being overweight at the age of ten years [96].

Antibiotics are a commonly administered, essential therapeutic agent used to treat bacterial infections. However, antibiotic exposure in early life can trigger intestinal dysbiosis through disruption of the commensal bacteria, in addition to harmful bacteria [99]. The impact of antibiotics on the microbiome depends on the duration, dosage, frequency, and age at the time of treatment [33]. The largest disruptions in the development of the microbiota occur when antibiotics are prescribed more frequently and in early life, which is also when antibiotic usage is most strongly correlated with becoming overweight [100]. Early-life antibiotic use can reduce the abundance of *Bifidobacterium*, the predominant colonizer of the neonatal gut, as well as increase the ratio of *Firmicutes*/*Bacteroidetes* [101,102]. Antibiotics have both transient and persistent effects on the gut microbiota and metabolic phenotype. Broad-spectrum antibiotic use significantly alters the gut microbiota composition and structure by reducing diversity by over 25% [102]. These effects, however, appear to be short-term and recover once treatment is completed [102]. A concerning long-term effect of antibiotic use is their potential to produce antibiotic-resistant genes within the gut [103]. Antibiotic-resistant genes can alter insulin sensitivity, elevate inflammatory cytokines and modify SCFA metabolism and bile acid production, all of which represent potential mechanisms of microbiota induced obesity [33]. 

It has been estimated that the average U.S. child, by the age of two, has already received three courses of antibiotics with that number rising to approximately eleven courses by the age of ten [104]. Antibiotic use in early life perturbs the colonization, composition, and maturation of the gut microbiota during a critical time, when the immune system, metabolic processes, and adipocytes are developing [101,105,106,107]. Children exposed to antibiotics during the first two years of life are at an increased risk of developing obesity [108]. In addition to postnatal antibiotic exposure, it has also been estimated that over 50% of women in the U.S. receive antibiotics during pregnancy [109]. Maternal antibiotic use during pregnancy and while breastfeeding has been associated with neonatal microbial dysbiosis [109,110,111]. Intrapartum exposure to antibiotics (i.e., antibiotics administered during labor and delivery) can reduce breast milk microbial diversity and decrease the number of *Bifidobacterium* that colonize the neonatal gut [112]. It has been theorized that maternal antibiotic use affects the fetal microbiota via several potential routes including the umbilical cord blood, during bacterial transmission from mother to fetus, or through the alteration of the vaginal microbes [101]. Both breast milk and maternal gut microbes are major factors that help lay the foundation for the initial colonization of the neonatal gut microbiota. 

## 4. Animal Studies Linking Early-Life Antibiotic Exposure to Obesity

Animal research is essential for understanding the underlying biological mechanisms that mediate the potential relationship between early life antibiotic use and obesity risk. Alterations to the gut microbiota during critical periods of development and maturation are postulated to be a critical player in this relationship (Table 1). A landmark study exploring the microbiota-centric nature of the antibiotic-obesity link was conducted by Cho et al. in 2012 [81]. Subtherapeutic antibiotic therapy (STAT) (i.e., penicillin, vancomycin, a combination of the two, chlortetracycline, or no antibiotics) was administered to young mice at weaning. STAT mice had significantly greater fat mass and percent body fat compared to control mice in the absence of increased chow intake. STAT mice showed an increased *Firmicutes*/*Bacteroidetes* ratio and increased abundance of *Lachnospiraceae* [81]. Metabolic changes occurred in the STAT microbiota with upregulation of genes involved in the metabolism of carbohydrates that were associated with increased fecal SCFA concentrations, potentially indicating increased energy extraction.

Following this pivotal demonstration that antibiotic administered to mice at weaning increases obesity risk, the Blaser group investigated whether earlier administration would have a greater effect. Cox et al. administered low dose penicillin to dams in the last week of gestation and throughout lactation [113]. Male offspring had accelerated growth prior to weaning and higher fat mass as adults. Maternal low-dose penicillin resulted in reduced levels of the satiety hormone, PYY and a trend towards elevated serum leptin in the mice. Importantly, the composition and structure of the microbiota were disrupted before a rise in adiposity was observed [113], suggesting that the microbiota played a role in the antibiotic-induced changes to body composition. Indeed, fecal microbiota transplant (FMT) from mice treated with low-dose penicillin to germ free mice was sufficient to increase total body weight and fat mass compared to control. An important observation in this study was that after antibiotic treatment stopped, the composition of the microbiota recovered but the metabolic phenotype remained [113]. 

Similar to what was shown in mice, Klancic et al. showed that early life STAT in Sprague–Dawley rats also increased obesity risk [114]. Low-dose penicillin was administered to pregnant dams began in the third week of pregnancy and continued throughout lactation. In contrast to other studies that largely focus on the offspring, Klancic et al. [114] showed that the dams receiving antibiotic had greater fat mass and fasting leptin and also had impaired post-partum weight loss. Fecal matter of antibiotic dams was enriched in *Enterobacteriaceae* and lower in *Lactobacillus* spp. The offspring of dams exposed to antibiotic had higher body weight, fat mass, and liver triglycerides, especially after being switched to an obesogenic high fat, high sucrose diet [114]. The antibiotic-associated obese phenotype was more prominent in the male versus female offspring. Post-weaning microbiota fecal analysis showed similar patterns in male offspring as dams, with reduced abundance of *Lactobacillus* spp. and increased abundance of *Enterobacteriaceae* observed. Although fecal microbiota transplant of adult male cecal microbiota could not transfer the obese phenotype to GF mice, there was a trend for increased weight gain [114]. 

Klancic et al. also examined post-natal antibiotic exposure in Sprague–Dawley rats using a pulsed antibiotic treatment with azithromycin [115]. Following three courses of a therapeutic dose of azithromycin and weaning onto a high fat/sucrose diet, an obese and insulin resistant phenotype developed, particularly in males [115]. Reduced hepatic expression of insulin receptor substrates and ileal tight junction proteins were plausible mechanisms identified to explain the insulin resistance. Importantly, in both Klancic’s maternal antibiotic exposure [114] and post-natal exposure study [115], prebiotic oligofructose, which is a known bifidogenic substrate, significantly attenuated the obese phenotype associated with antibiotic exposure. 

In another post-weaning study using a piglet model, low-dose antibiotic (chlortetracycline and virginiamycin) administration resulted in a significantly increased growth rate [116]. Using PICRUSt (phylogenetic investigation of communities by reconstruction of unobserved states) to infer microbial function, the authors show that the carbohydrate metabolism pathway was significantly elevated in microbiota from antibiotic exposed piglets [116]. The microbial community that contributes to SCFA production was enriched in response to the antibiotic, suggesting the growth-promoting effect of the antibiotic could be related to better energy utilization.

In a unique co-housing experiment, Schulfer et al. showed that co-housing control mice with mice with a STAT-perturbed microbiota could partially reduce body weight in the STAT mice [117]. The weight-reducing effect of co-housing with a healthy animal was only seen when the mice consumed chow and switching to an obesogenic high-fat diet eliminated the protective effect of co-housing [117], suggesting that diet can have a profound effect on microbial and metabolic responses. Further mechanistic insight into the effects of early-life antibiotic exposure on obesity has been provided by Nobel et al. [118]. In a study using a post-weaning pulsed antibiotic treatment with therapeutic doses of amoxicillin (a beta-lactam) or tylosin (a macrolide), both antibiotics were shown to enrich for genes associated with the synthesis of the endotoxin lipopolysaccharide, which has been associated with chronic low-grade inflammation and obesity [118]. 

In contrast to the numerous animal studies demonstrating an obesogenic effect of early-life antibiotic exposure, two studies in rats did not find an accompanying increase in weight or growth [119,120]. In the first instance, rats were exposed to antibiotic (amoxicillinum trihydricum) either pre-weaning or post-weaning while controlling the litter size to prompt overnutrition (small litter of four) or normal nutrition (large litter of ten) [120]. While rats from small litters had increased weight gain, the antibiotic did not have any significant effect on weight gain, fat mass, or food intake. In the second instance, rats were exposed to high doses of amoxicillin during suckling and despite a transient increase in food intake, there were no significant effects on body weight, weight gain, fat mass, or lean mass [119]. Given that high doses of antibiotic have been previously attributed to weight loss [99], it is possible some of the contrasting outcomes are related to differences in antibiotic dose, class, number of courses, duration, and age of administration. 

Findings from the majority of experimental animal studies are in agreement with the corresponding human observational research (reviewed next). The type of antibiotics administered, the dose, the number of courses, and the timing of exposure are likely factors affecting the outcomes of antibiotic use. The evidence supports the theory that the development of the microbiota in early life programs long term metabolic function. Perturbations to the normal development of the gut microbiota via antibiotics can alter the microbiota, often only transiently, but the metabolic consequences can be long-lasting particularly when animals encounter an obesogenic diet, such as high fat/high sucrose.

## 5. Human Studies Linking Early-Life Antibiotic Exposure to Obesity

Early-life antibiotic exposure has the potential to alter gut microbiota and in turn increase the propensity to develop obesity later in life. Observational studies examining early life antibiotics range from exposure during a defined period, such as prenatal exposure to lifetime exposure up until the age of seven. In general, the scientific literature that simultaneously explores antibiotic use, alterations to the gut microbiota, and later obesity risk is quite limited. There are, however, some consistencies within the available evidence that suggest childhood obesity risk and infant microbiota alterations following antibiotic treatment are linked (Table 2).

Across an number of studies, a consistent association has been found between antibiotic-induced changes in the gut microbiota and either childhood adiposity or obesity [121,122,123,124,125]. Zhang and colleagues investigated the influence that prenatal antibiotic exposure had on infant weight for length score (WFL-score), adiposity, and alterations in the gut microbiota [121]. A total of 237 of the 454 infants included in the study were exposed to antibiotics during gestation, which, for the purposes of the study, was defined as second-trimester exposure. Stool samples were collected at three and twelve months postpartum. Amplicon sequence variants (ASVs) analysis showed that the abundance of 13 bacterial ASVs at three months and 17 ASVs at twelve months varied between control infants and those exposed to antibiotic. *Lachnospiraceae* was enriched in infants exposed to prenatal antibiotics at both three months and twelve months postpartum. In a Canadian cohort, *Lachnospiraceae* abundance in infancy was associated with increased odds of overweight or obesity at 3 years of age [126]. 

Chen et al. examined the effect of antibiotic use in the first year of life in a mother-offspring cohort from Singapore and found that antibiotic treatment was associated with altered gut microbiota composition, increased adiposity, and elevated risk of childhood obesity [122]. Stool samples collected at 24 months of age showed that repeated antibiotic use reduced the microbial co-abundant group (CAG) represented by *Eubacterium hallii*, which was negatively correlated with childhood adiposity. The CAGs represented by *Tyzzerella 4* and *Gemella* was also amplified and was positively correlated with childhood adiposity. Any exposure to antibiotic in the first year was associated with a higher odds of obesity from 15 to 60 months of age (OR(95% CI) = 1.45(1.001, 2.14)) [122]. 

Korpela and colleagues investigated lifetime antibiotic use, BMI, and gut microbiota in Finnish children in a series of studies [123,124,125]. In one study, fecal samples from 162 Dutch and Finnish children at 3 months of age were examined to determine if BMI at 5–6 years of age was associated with microbiota composition and if antibiotic treatment altered this relationship [123]. *Bifidobacterium* was negatively associated with BMI while streptococci were positively associated with BMI, although the associations varied by antibiotic exposure and were only seen when children received several courses of antibiotics.

In a second study by this group, lifetime macrolide treatment was compared to penicillin treatment in Finish children between the ages of two to seven years [124]. Macrolide use but not penicillin use was associated with a compositional shift in microbiota with decreased *Actinobacteria* and increased *Bacteroidetes* and *Proteobacteria*. Although phylum level shifts largely resolved within one year after macrolide use, there was a long-term reduction in microbial richness. The positive correlation seen between macrolide use and BMI z-score suggests that even transient disturbances to the microbiota in early life could have long-lasting effects.

In a third study with the Finnish cohort, Korpela et al. examined whether antibiotic use in a child during breastfeeding interfered with the protective effect of breastfeeding against risk of overweight [125]. In children who did not receive antibiotics in early life, breastfeeding duration was negatively associated with BMI z-score. No association was shown among children who did receive antibiotics [125]. The abundance of *Bifidobacterium* was reduced in children with short duration of breastfeeding or early-life antibiotic use.

The relationship between antibiotics and obesity appears to depend on the number of courses administered [121,122,123,124]. After controlling for potential confounders, Zhang and colleagues found that a greater abdominal skinfold thickness at twelve months was observed when participants were exposed to ≥ three courses of prenatal antibiotics [121]. Chen et al. reported that any antibiotic use during the first year of life was associated with increased obesity risk at 15–60 months of age, but that relationship was strongest in boys who received ≥3 courses [122]. Macrolide use was strongly correlated with childhood BMI when two courses of macrolides were used within the first two years of life [124]. 

The association between obesity and antibiotics may also be influenced by the timing of administration. Zhang et al. reported that the strongest relationship between prenatal antibiotic exposure and elevated weight-for-length-score at twelve months of age occurred when antibiotic exposure happened during the second trimester [121]. Macrolide treatment before the age of two disturbed the gut microbiota and led to long-lasting metabolic changes associated with obesity [124]. 

The evidence to date suggests that antibiotic exposure in early life can cause persistent and significant alterations to an infant’s gut microbiota. Some of the observed changes in gut microbiota have been linked with altered metabolic function, increased adiposity, and obesity in childhood. The reported findings are not causal, thus the underlying mechanism linking early life antibiotic treatment, obesity, and the gut microbiota are not fully understood. The research simultaneously investigating early-life antibiotic use on gut microbiota dysbiosis and obesity in humans is limited and currently lacking with regards to risks into adulthood. Further research is warranted to understand the long-term effects of early-life antibiotics, particularly investigating adulthood obesity and persistent changes to the gut microbiota and metabolism. 

## 6. Potential Interventions to Mitigate Antibiotic-Associated Obesity Risk

As our understanding of the impact of antibiotics on gut microbiota and metabolic health increases, there is a growing interest in identifying potential therapeutic interventions that could mitigate the adverse consequences of antibiotics. Modification of the gut microbiota is likely the primary mechanism causing antibiotic-induced obesity; as a result, the therapeutic interventions investigated to date target the composition of the gut microbiota. 

Diet is a key determinant of gut microbiota composition [39,127]. The quality and quantity of dietary fats consumed alter gut microbiota composition, with omega-3 fatty acids in particular showing a positive impact on the gut microbiota [128]. This has been eloquently demonstrated in fat-1 mice that endogenously produce omega-3 fatty acids [129]. Fat-1 mice exposed to three intermittent courses of azithromycin following by a high-fat diet supplemented with omega-3 fatty acids displayed favorable increases in the abundance of *Bifidobacterium* and decreases in *Enterobacteriaceae* [130]. Elevated endogenous production of omega-3 fatty acids in the mice counteract early life antibiotic-induced dysbiosis, as well as reduced body weight and insulin resistance severity [130]. Part of the protective effects of omega-3 fatty acids appear to involve an anti-inflammatory effect, whereby it was shown that mice consuming a diet high in omega-6 fatty acids had higher levels of serum LPS and pro-inflammatory cytokines, while fat-1 mice had dramatically reduced LPS and low-grade inflammation [131]. 

The altered gut microbiota and LPS production observed with increased endogenous omega-3 fatty acids was linked to a concomitant increase in intestinal alkaline phosphatase (IAP) production and secretion within the intestine [131]. IAP is an intestinal brush border enzyme that is known to maintain normal gut microbial homeostasis, inhibit the growth of certain pathogenic bacteria, and detoxify LPS [131,132]. When IAP was co-administered with azithromycin to mice in early life followed by a high-fat diet, IAP was able to normalize body weight, serum lipids and glucose levels to that of control mice and restore normal commensal microbiota [133]. 

Prebiotics are another dietary strategy that show promise as a means to favorably manipulate the gut microbiota and improve metabolic health [134]. Prebiotics are defined as “a substrate that is selectively utilized by host microorganisms conferring a health benefit” [75]. The current definition expands prebiotic substrates beyond the traditionally recognized oligosaccharides (e.g., inulin, fructooligosaccharides) to include non-carbohydrate-based substances (e.g., phenolics and phytochemicals). Prebiotics (i.e., oligofructose and inulin) have been shown to reduce body fat in children [135] and adults [136] with overweight and obesity. Co-administration of prebiotic oligofructose with low-dose penicillin to rat dams during pregnancy and lactation, significantly decreased fat mass in their offspring and increased the abundance of *Bifidobacterium* in dams and their offspring at weaning [114]. In addition to the protection of oligofructose consumed by the mothers, direct consumption of oligofructose by young rats also reduced obesity risk associated with exposure to three pulses of a therapeutic dose of azithromycin [115]. Insulin resistance induced by antibiotic was reversed with prebiotic co-administration in male rats [115]. Other prebiotics beyond the classical oligosaccharides warrant examination in future studies.

When antibiotic use is clinically necessary during critical periods of life, especially during pregnancy and the first year of life, co-administration of omega-3 fatty acids, IAP, or prebiotics are promising therapeutic interventions that warrant further investigation, particularly randomized controlled trials for their ability to prevent gut dysbiosis and later life obesity. 

## 7. Conclusions

Animal and human studies suggest that gut microbiota plays a key role in the association seen between antibiotics and obesity risk. It was previously estimated that approximately 50% of antibiotic prescriptions are unnecessary [101]; therefore, a greater understanding of the long-term metabolic consequences of antibiotic use, particularly in early life, may lead to greater antibiotic stewardship. When antibiotic treatment is clinically necessary, omega-3, IAP, and prebiotic supplementation represent interventions currently being investigated for their potential to mitigate the adverse effects antibiotics have on the gut microbiota.

## Figures and Tables

**Table 1:** Animal studies linking early-life antibiotic exposure to obesity risk.

Study	Model	Antibiotic Use and Age of Administration	Outcome
Cho et al. [81]	C57BL/6J mice	Subtherapeutic antibiotic therapy (STAT) (penicillin, vancomycin, a combination of the two, or chlortetracycline); weaning	Greater fat mass; increased *Lachnospiraceae*; increased fecal short-chain fatty acid (SCFA) concentrations
Cox et al. [113]	C57BL/6J mice	Low-dose penicillin; maternal during last week of gestation and during lactation	Male offspring accelerated early growth; greater fat mass as adults; reduced satiety hormone peptide tyrosine tyrosine (PYY); lower *Lactobacillus*; acceleration of normal age-related microbiota development
Klancic et al. [114]	Sprague–Dawley rats	Low-dose penicillin; maternal during last week of gestation and during lactation	Impaired post-partum weight loss in dams; lower *Lactobacillus* and higher *Enterobacteriaceae* in dams; higher body fat and liver triglycerides in offspring; lower *Lactobacillus* in offspring at weaning; impairments reversible with prebiotic oligofructose co-administration
Klancic et al. [115]	Sprague–Dawley rats	Pulsed therapeutic doses of azithromycin; postnatal days 19–21, 28–30, 37–39	Higher body weight in male and female offspring at 10 weeks of age; higher fat mass and insulin resistance in males; reduced *Lactobacillaceae* in males and females; prebiotic oligofructose co-administration corrected insulin resistance
Che et al. [116]	Piglets	Low-dose chlortetracycline and virginiamycin; post-weaning	Increased growth rate and food intake; enriched *Methanosphaera* species; enrichment in carbohydrate metabolism pathway
Schulfer et al. [117]	C57BL/6J mice	STAT penicillin G; maternal during pregnancy through to 4 weeks of age of pups	Co-housing unified microbiota within cages lowering weight of STAT mice relative to non-cohoused mice; co-housing could not attenuate impact of STAT once mice switched to a high fat diet
Nobel et al. [118]	C57BL/6J mice	Pulsed therapeutic doses of amoxicillin or tylosin; post-weaning	Early growth acceleration; tylosin increased hepatic steatosis; enrichment of genes for lipopolysaccharide synthesis
Morel et al. [119]	Sprague–Dawley rats	High dose amoxicillin; suckling period postnatal day 5 to 15	Transient increase in food intake, but no effect on body weight or fat mass
Mozes et al. [120]	Sprague–Dawley rats	Amoxicillinum trihydricum; maternal up to 15 days lactation and post-weaning from day 21 to day 40 in small and normal litter sizes	Small litters had increased weight gain, which was not influenced by antibiotic treatment; *Lactobacillus* decreased with antibiotic

**Table 2:** Human studies linking early-life antibiotic exposure to obesity risk.

Study	Participants/Subjects	Antibiotic Use and Age of Administration	Outcome
Zhang et al. [121]	454 mother-offspring dyads from Southeastern USA; singleton infants born ≥28 weeks gestation with no congenital abnormalities	Beta-lactam antibacterials, penicillins; other beta-lactam antibacterials; sulfonamides and trimethoprim; macrolides, lincosamides, streptogramins; aminoglycoside antibacterials; and other antibacterials; maternal antibiotic use in 1st, 2nd, 3rd trimester and 1st year after delivery; and infant antibiotic use in first year of life	Second trimester antibiotic exposure associated with higher weight-for-length score at 12 months; 13 bacterial amplicon sequence variants (ASVs) at 3 months, and 17 ASVs at 12 months varied between control and antibiotic exposed
Chen et al. [122]	1172 Mother-offspring dyads from Singapore cohort of Chinese, Malay or Indian ethnicity; singleton infants	Antibiotic type not investigated; infant antibiotic exposure in the first year of life	Elevated risk of childhood obesity for any antibiotic exposure; higher body mass index (BMI) z-score in boys but not girls; reduced *Eubacterium hallii*; higher *Tyzzerella 4* was positively correlated with childhood adiposity and repeated antibiotic use
Korpela et al. [123]	162 vaginally born Dutch and Finnish children	Antibiotic type not investigated; lifetime antibiotic use to 5–6 years of age	After several courses of antibiotics low relative abundance of *Actinobacteria* and high relative abundance of *Firmicutes* at 3 months of age associated with high BMI at 5–6 years
Korpela et al. [124]	236 Finnish children aged 2–7 years	Penicillin; macrolides; lifetime antibiotic use	Macrolides use decreased *Actinobacteria* and increased *Bacteroidetes* and *Proteobacteria*; long-term reduction in microbial richness with macrolide use; >2 courses of macrolides in first 2 years of life strongly correlated with childhood BMI
Korpela et al. [125]	226 Finnish children aged 2–6 years	Penicillin-type antibiotics; lifetime antibiotic use	Breastfeeding duration negatively associated with BMI z-score in children with no early-life antibiotic use; with early-life antibiotics the duration of breastfeeding was no longer associated with BMI z-score; early-life antibiotic use associated with reduced *Bifidobacterium* abundance
Tun et al. [126]	757 infants from Canadian cohort; singleton birth at ≥35 weeks of gestation with a birth weight of ≥2500 g	Cleaning product use but also maternal intrapartum antibiotic prophylaxis; infant antibiotic treatment by 3 months	Prevalence of overweight at 3 years higher after intrapartum antibiotic prophylaxis and infant antibiotic treatment; higher *Lachnospiraceae* abundance in infancy increased odds of overweight at 3 years of age
